# Hepatoprotective Effect of *Lactiplantibacillus plantarum* DSR330 in Mice with High Fat Diet-Induced Nonalcoholic Fatty Liver Disease

**DOI:** 10.4014/jmb.2310.10026

**Published:** 2023-12-08

**Authors:** Na-Kyoung Lee, Yunjung Lee, Da-Soul Shin, Jehyeon Ra, Yong-Min Choi, Byung Hee Ryu, Jinhyeuk Lee, Eunju Park, Hyun-Dong Paik

**Affiliations:** 1 Department of Food Science and Biotechnology of Animal Resources, Konkuk University, Seoul 05029, Republic of Korea; 2 Department of Food and Nutrition, Kyungnam University, Changwon 51767, Republic of Korea; 3 FM MI center, Daesang Wellife, Seoul 03130, Republic of Korea; 4 Jongga R&D product Division, Daesang, Seoul 03130, Republic of Korea

**Keywords:** Nonalcoholic fatty liver, hepatoprotective effect, probiotics, *Lactiplantibacillus plantarum*, animal model

## Abstract

*Lactiplantibacillus plantarum* DSR330 (DSR330) has been examined for its antimicrobials production and probiotics. In this study, the hepatoprotective effects of DSR330 were examined against non-alcoholic fatty liver disease (NAFLD) in a high-fat diet (HFD)-fed C57BL/6 mouse model. To induce the development of fatty liver, a HFD was administered for five weeks, and then silymarin (positive control) or DSR330 (10^8^ or 10^9^ CFU/day) was administered along with the HFD for seven weeks. DSR330 significantly decreased body weight and altered serum and hepatic lipid profiles, including a reduction in triglyceride, total cholesterol, and low-density lipoprotein cholesterol levels compared to those in the HFD group. DSR330 significantly alleviated HFD-related hepatic injury by inducing morphological changes and reducing the levels of biomarkers, including AST, ALT, and ALP. Additionally, DSR330 alleviated the expression of SREBP-1c, ACC1, FAS, ACO, PPARα, and CPT-1 in liver cells. Insulin and leptin levels were decreased by DSR330 compared to those observed in the HFD group. However, adiponectin levels were increased, similar to those observed in the ND group. These results demonstrate that *L. plantarum* DSR330 inhibited HFD-induced hepatic steatosis in mice with NAFLD by modulating various signaling pathways. Hence, the use of probiotics can lead to hepatoprotective effects.

## Introduction

The liver plays a major role in whole-body lipogenesis, cholesterol metabolism, and gluconeogenesis [1]. However, non-alcoholic fatty liver disease (NAFLD) is associated with increased accumulation of hepatocellular lipids [2]. NAFLD leads to abnormal liver function and can progress to more severe liver diseases such as nonalcoholic steatohepatitis (NASH), fibrosis, and cirrhosis [3]. The process of NAFLD involves two key stages: 1) Excessive accumulation of triglycerides (TG) within hepatocytes; 2) An increase in pro-inflammatory adipokines and cytokines, including tumor necrosis factor (TNF)-α and interleukin (IL)-6, accompanied by mitochondrial dysfunction and oxidative stress [4].

The AMP-activated protein kinase (AMPK) signaling pathway plays a crucial role in maintaining cellular energy metabolism [5]. AMPK, which facilitates lipogenesis and fatty acid oxidation in the liver, has become a major target for NAFLD therapy or prevention of NAFLD [6]. AMPK activation mediates energy metabolism by suppressing acetyl-CoA carboxylase (ACC) and fatty acid synthase (FAS) expression via decreased transcriptional activation of sterol regulatory element-binding protein (SREBP)-1c [7, 8]. Activation of ACC upon stimulation of AMPK results in reduced malonyl-CoA synthesis, which reduces fatty acid synthesis and increases mitochondrial fatty acid oxidation via the regulation of carnitine palmitoyltransferase (CPT)-1 [9, 10].

Efforts to develop hepatoprotective or therapeutic agents for liver diseases have predominantly focused on exploring medicinal herbs or combinations of herbs [11]. Recently, probiotics have been reported to exert a protective effect on the gut–liver axis [12]. Probiotics have been used as nutritious ingredients for the treatment of diseases, such as colon inflammation and neuronal disorders [13, 14]. Probiotics influence the gut microbiota and their metabolites, such as short-chain fatty acids (SCFAs). Some probiotics influence liver enzymes, lipid metabolism, blood glucose-related indices, body mass, and inflammation in patients [15]. VSL#3, which consists of eight probiotic strains [16] comprising *Lactiplantibacillus plantarum* ZJUIDS14 [17], and *Lactobacillus* sp. [18, 19] can effectively decrease TG and total cholesterol (TC) levels in the serum and the potency of the inflammatory response. Hence, it can be used as an adjuvant treatment of NAFLD.

*L. plantarum* DSR330, isolated from Korean fermented foods, has probiotic properties, including resistance to gastric conditions and antimicrobial effects [20]. The objective of this study was to investigate the preventive effects against NAFLD of *L. plantarum* DSR330 in high-fat diet (HFD)-fed mice and to obtain functional insights into the role of lactic acid bacteria (LAB) in preventing NAFLD.

## Materials and Methods

### Preparations of Bacterial Samples

*L. plantarum* DSR330 (DSR330, KFCC 11393P) was isolated from kimchi in Korea [20]. DSR330 was cultured as a probiotic in general media. The cultured strain was centrifuged and resuspended in PBS (HyClone, USA) or 1% glucose. The harvested strain was lyophilized and used for the development of animal models.

### Animal Groups and Experimental Design

Four-week-old, male C57BL/6 mice were purchased from Koatech (Republic of Korea). Mice were housed 3–4 per cage at 23 ± 2°C and 53 ± 2% relative humidity with a 12-h light/dark cycle. After one week of acclimatization, mice were randomly assigned to five groups (n=8) as follows: (1) normal-food diet (ND, 10% kcal fat), (2) HFD, high fat diet (60% kcal fat), (3) HFD with silymarin (Silymarin), (4) HFD with 10^8^ CFU/day of DSR330 (DSR-8), and (5) HFD with 10^9^ CFU/day of DSR330 (DSR-9). To induce the development of fatty liver, the HFD was administered for five weeks, and then silymarin (200 mg/kg) or DSR330 (10^8^ or 10^9^ CFU/day) was administered along with the HFD for an additional seven weeks. The body weight and food intake were measured weekly. At the end of the experimental period, all mice were subjected to fasting for 12 h, and their livers were collected for further analysis. All experimental protocols were performed in accordance with the guidelines of the Institutional Animal Care of Kyungnam University (KUICA-22-09).

After 12 weeks, the experimental animals were subjected to fasting for 12 h and anesthetized with isoflurane (4 ml/kg). Blood was collected and centrifuged at 2,000 ×*g* for 30 min to separate the serum. After blood collection, the organs were harvested and evaluated. All samples were stored at –80°C until further analysis.

### Histological Analysis of Liver Samples

The liver tissues were fixed in 10% paraformaldehyde for 24 h. Subsamples of hepatic tissues were embedded in paraffin. The tissues were cut into 4-μm-thick sections, stained with hematoxylin and eosin (H&E), and evaluated by light microscopy.

### Biochemical Analysis of Serum and Liver Samples

Serum levels of triglyceride (TG), total cholesterol (TC), high-density lipoprotein cholesterol (HDL), low-density lipoprotein cholesterol (LDL), alanine transaminase (ALT), alkaline phosphatase (ALP), and aspartate transaminase (AST) were determined using a determination kit (BioSystems, Spain) according to the manufacturer’s guidelines.

### RNA Extraction and Quantitative Real-Time Polymerase Chain Reaction (PCR)

Total RNA was extracted from the liver and subcutaneous adipose tissue using the TRIzol Reagent (Invitrogen, USA). The cDNAs were synthesized from 1 μg of RNA using M-MLV reverse transcriptase (Promega, USA). After cDNA synthesis, quantitative real-time PCR was performed using 25 μl of SYBR Green master mix (PhileKorea, QuantiSpeed SYBR No-ROX kit, Korea) with Real-time DNA thermal cycler (CFX Duet real-time PCR system, Bio-Rad, USA). The reaction mixtures were incubated for initial denaturation at 95°C for 10 min, followed by 50 cycles of PCR. Each cycle was performed as per the following parameters: 95°C for 10 s, 55°C for 20 s, and 72°C for 20 s. The sequences of the sense and antisense primers used for amplification are listed as follows: PPARγ, sense, 5'-ccacactatgaagacattccat-3' and antisense, 5'-gttctactttgatcgcactttg-3'; SREBP-1c, sense, 5'-gtgtgcaccgtagttctggg-3' and antisense, 5'-aggtcagcttgtttgcgatg-3'; ACC1, sense, 5'-ccctacacttactgatgagc-3' and antisense, 5'-gggaagcaataagaacctga- 3'; FAS, sense, 5'-aagaaagtgctggaaaagga-3' and antisense, 5'-cagcaattctcgggatgtat-3'; ACO, sense, 5'-attaagtcgccaccattctt-3' and antisense, 5'-ggtccgttgttactgaatct-3'; PPARα, sense, 5'-gaatccacgaagcctacc-3' and antisense, 5'-gccatacacaaggtatcc- 3'; CPT-1, sense, 5'-aagatcaatcggaccctaga-3' and antisense, 5'-atagtcatgatgatcgaaac-3'. The β-Actin-encoding gene was used as a reference gene. The normalized target gene expression levels in the sample were calculated using 2ΔΔCT. Values were expressed as fold change compared to control and are represented as the mean ± SE (*n* = 7).

### Analysis of Hormones Related to Energy Metabolism

Serum insulin, adiponectin, and leptin levels were measured using a microplate reader (Epoch, BioTek Instruments Inc., USA) with an ELISA kit (BioVendor R&D, Czech).

### Statistical Analysis

All data are presented as the mean ± standard error. One-way analysis of variance and Duncan’s multiple range test were used to compare multiple groups. The results were considered statistically significant at *p* < 0.05, and all statistical analyses were conducted using the SPSS software (IBM, USA).

## Results

### Reduction in Body Fat and Liver Weight in Mice with NAFLD Mediated by DSR330

**Fig. 1:**
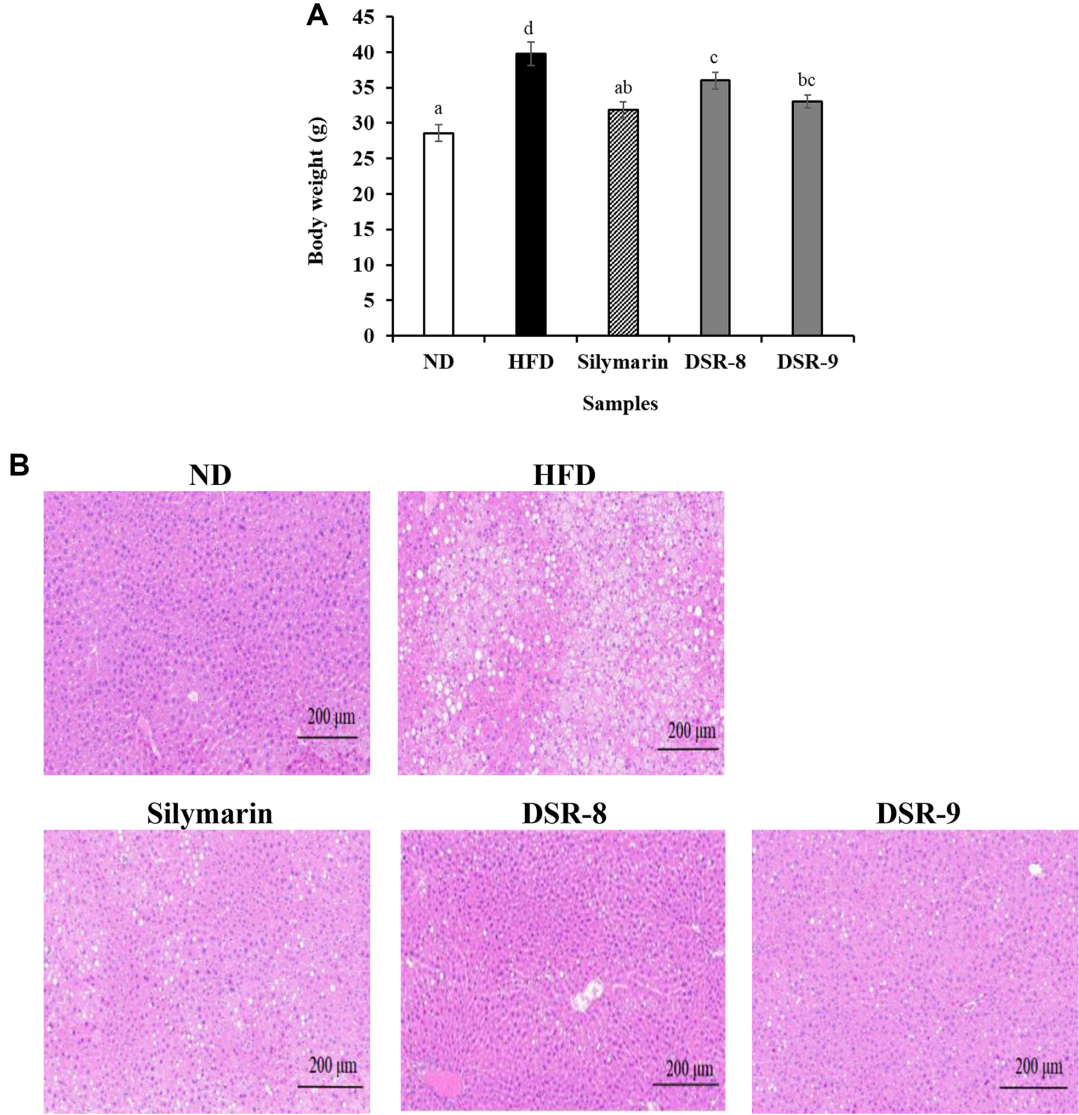
Effects of *Lactiplantibacillus plantarum* DSR330 on HFD-fed mice (*n* = 8). (**A**) Body weight, (**B**) H&Estained liver tissue. Data are presented as mean ± standard error of triplicate experiments. Different letters on the error bars represent significant differences (*p* < 0.05). ND, normal-food-diet (10% kcal fat); HFD, high-fat-diet (60% kcal fat); Silymarin, HFD with silymarin; DSR-8, HFD with 10^8^ CFU/day of DSR330; DSR-9, HFD with 10^9^ CFU/day of DSR330.

The HFD was administered for five weeks to induce the development of fatty liver in the NAFLD mouse model. After five weeks, silymarin and DSR were administered to treat NAFLD for 12 weeks. Body weight and body fat are presented in Fig. 1A and Table 1. The body weights of the mice in each group showed an increasing trend. The body weight of the HFD group (39.58 ± 1.98 g) increased significantly compared with the ND group (29.43 ± 1.31 g). The body weight was significantly reduced after administration of silymarin (32.50 ± 1.30 g), DSR-8 (36.93 ± 1.53 g), and DSR-9 (33.86 ± 1.09 g) treatment for 12 weeks. Liver weight in silymarin- and DSR-8-treated groups was reduced by 79.10% (2.46 ± 0.38 g/100 g BW) and 84.57% (2.63 ± 0.11 g/100 g BW), respectively, compared to the HFD (3.09 ± 0.64 g/100 g BW) group (data not shown). The HFD increased hepatic steatosis, as determined by hematoxylin and eosin staining (Fig. 1B). The fatty livers showed degermation and the formation of lipid droplets in the HFD group, whereas the livers showed decreased variations in the DSR groups (Fig. 1B). The body fat was characterized as brown, subcutaneous, and visceral (Table 1). HFD increased subcutaneous fat and visceral fat compared to ND. The administration of silymarin and DSR-9 led to notable variations in subcutaneous fat within this dataset.

### Alleviatory Effects of DSR330 on Hepatic Steatosis and Liver Damage in Mice with NAFLD

**Fig. 2:**
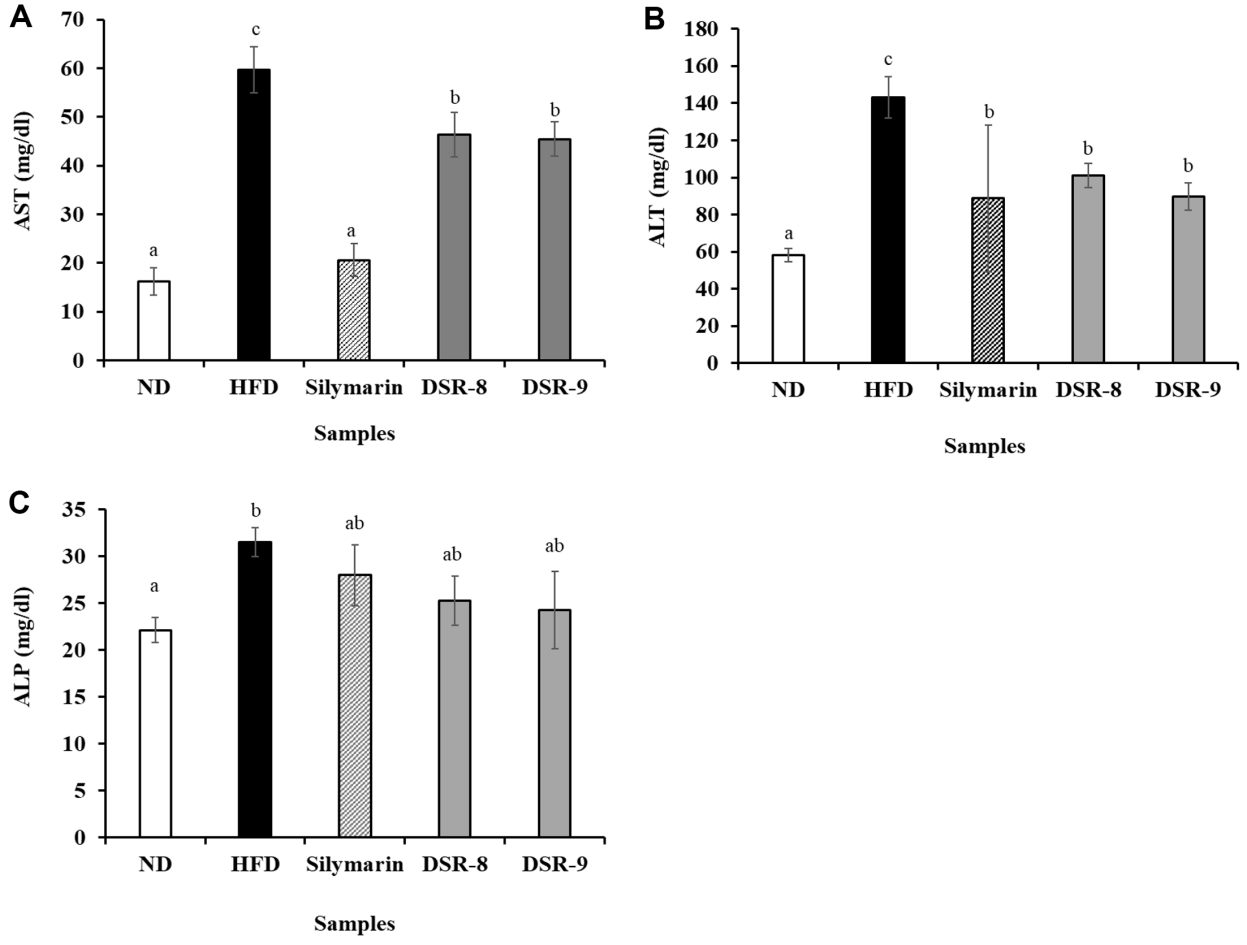
Effects of *Lactiplantibacillus plantarum* DSR330 on hepatic steatosis in the liver of HFD-fed mice (*n* = 8). (**A**) AST, (**B**) ALT, and (**C**) ALP levels. Data are presented as mean ± standard error of triplicate experiments. Different letters on the error bars represent significant differences (*p* < 0.05). ND, normal-food-diet (10% kcal fat); HFD, high-fat-diet (60% kcal fat); Silymarin, HFD with silymarin; DSR-8, HFD with 10^8^ CFU/day of DSR330; DSR-9, HFD with 10^9^ CFU/day of DSR330.

Assessment of liver function-related enzymes and an increase in AST, ALT, and ALP in serum can help detect damage to hepatocytes and the biliary tract [21, 22]. The results of Fig. 2 represent the liver damage based on levels of biomarkers in the serum. The values of AST (3.69-fold), ALT (2.46-fold), and ALP (1.43-fold) in the HFD group increased compared to those in the ND group (Fig. 2A-C). DSR decreased AST (0.76~0.78-fold), ALT (0.62~0.71-fold), and ALP (0.77~0.80-fold) levels compared to those observed in the HFD group. DSR was more effective than silymarin with respect to reduction in ALP levels.

### Effects of DSR330 on Serum Lipids in Mice with NAFLD

**Fig. 3:**
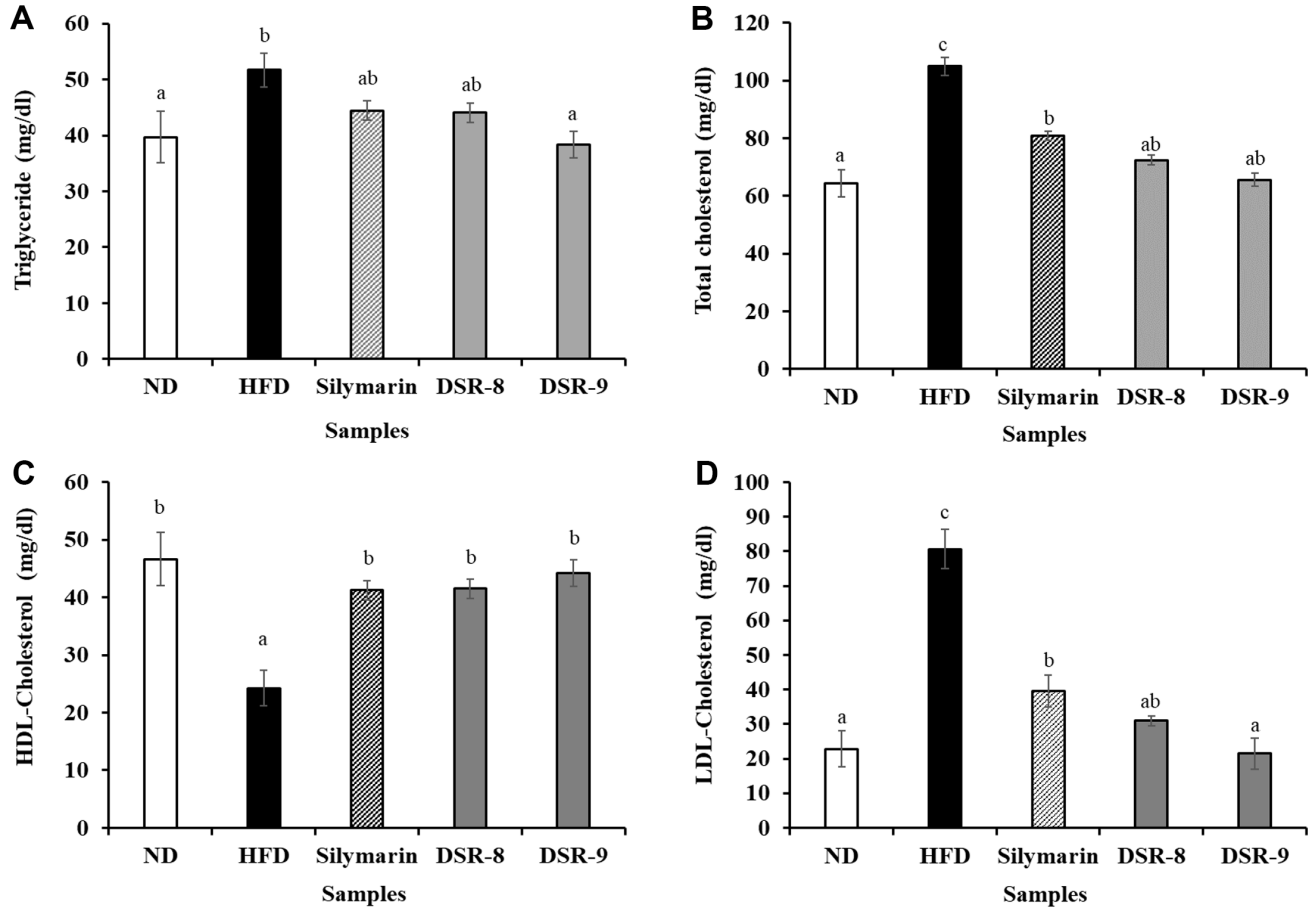
Effects of *Lactiplantibacillus plantarum* DSR330 on serum lipid profiles of HFD-fed mice (*n* = 8). (**A**) Triglyceride, (**B**) total cholesterol, (**C**) HDL-cholesterol, and (**D**) LDL-cholesterol levels. The data are presented as mean ± standard error of triplicate experiments. Different letters on the error bars represent significant differences (*p* < 0.05). ND, normal-food-diet (10% kcal fat); HFD, high-fat-diet (60% kcal fat); Silymarin, HFD with silymarin; DSR-8, HFD with 10^8^ CFU/ day of DSR330; DSR-9, HFD with 10^9^ CFU/day of DSR330.

Fig. 3 shows the serum lipid variables in mice with NAFLD. Serum TG, TC, and LDL levels in the HFD group increased by 130.2% (51.69 ± 3.07 mg/dl), 162.83% (104.93 ± 6.46 mg/dl), and 353.04% (80.67 ± 5.75 mg/dl) compared to those of the ND group, respectively. DSR-9 significantly reduced these levels by 74.23%(38.37 ± 2.35 mg/dl), 62.55% (65.63 ± 3.77 mg/dl), and 26.59% (21.45 ± 4.41 mg/dl), respectively. The DSR-9 group exhibited similarity to the ND group concerning all the measured variables. In addition, the level of HDL-cholesterol increased by 182.11% (44.18 ± 1.29 mg/dl) compared to that in the HFD (24.26 ± 1.45 mg/dl) group, and these values were similar to those observed in the ND group. In particular, DSR was more effective than silymarin in reducing the levels of serum lipids.

### Effects of DSR330 on Lipid Synthesis, Lipolysis, and Fatty Oxidation in the Liver of Mice with NAFLD

The mRNA expression of PPARγ and SREBP-1c, two key transcription factors regulating lipid synthesis, was increased in the HFD group (Table 2). However, DSR treatment decreased SREBP-1c expression. In addition, DSR reduced the levels of ACC1 and FAS, which are downstream targets of PPARγ and SREBP-1c. ACO, PPARα, and CPT-1 are related to fatty acid oxidation and adiponectin production (Table 3). While the HFD led to a decrease in these factors, both the DSR and the ND groups showed an increase, similar to the silymarin group.

### Effect of DSR330 on Insulin, Adiponectin, and Leptin Levels in Mice with NAFLD

The data collected after the evaluation of metabolic hormones are presented in Table 4. The insulin and leptin levels were higher in the HFD group than in the ND group. However, silymarin, DSR-8, and DSR-9 treatments decreased the secretion of these hormones. Adiponectin levels decreased in the case of increased HFD treatment. Silymarin, DSR-8, and DSR-9 treatments exhibited similarity to the ND group, indicating a prophylactic effect on fatty liver in NAFLD mice.

## Discussion

This study demonstrated that the administration of DSR330 significantly alleviated metabolic disorders in HFD-fed mice. NAFLD is characterized by the accumulation of fat droplets in hepatocytes [4] and an increase in liver weight and TG levels. DSR330 reduced liver weight and TG levels, suggesting that DSR330 was well able to alleviate steatosis. H&E staining of liver tissues revealed liver steatosis and vacuolar degeneration in the HFD group, suggesting that HFD induced histopathological damage. However, treatment with DSR330 alleviated liver dysfunction and damage, which was further confirmed by changes in the AST, ALT, and ALP levels.

Obesity can lead to dysbiosis of the gut microbiota via disruption of the intestinal barrier, including tight junction proteins (claudins and ZO-1), the mucus layer (Muc2), and IgA secretion [23]. These conditions can be attributed to metabolic diseases, including type 2 diabetes mellitus, cardiovascular disease, NAFLD, and hypertension [24, 25]. *L. plantarum* MGEL20154 has shown anti-obesity and probiotic effects [24]. In addition, medicinal plants, such as blue honeysuckle, have shown anti-obesity and fatty liver preventive effects [25].

Serum AST, ALT, and ALP levels are major enzymes present in hepatocytes, and their levels increase following hepatocellular injury [26]. Among these biomarkers, ALT is particularly sensitive and closely associated with NAFLD [27]. In many clinical studies examining NAFLD, increased ALT levels have been considered independent predictors [28]. Probiotics can modify gut dysbiosis caused by obesity, leading to anti-inflammatory effects under inflammatory conditions [13].

Thus, obesity-induced liver damage can be alleviated, leading to a reduction in ALT and AST levels after the administration of probiotics [23]. DSR330 significantly decreased the HFD-induced elevation in serum AST, ALT, and ALP levels in the liver tissue of HFD-fed mice (Fig. 2). Moreover, histopathological observation of liver tissues by H&E staining showed that DSR330 markedly attenuated the excessive formation and accumulation of lipid droplets in hepatocytes.

DSR330 downregulated lipid metabolism related to lipogenesis and lipid oxidation in the liver of HFD-fed mice (Tables 2 and 3). In addition, DSR330 was able to predict the potential activation of the AMPK signaling pathway related to fatty acid oxidation in the liver. In obesity mouse model, upregulation of AMK can alleviate fatty liver disease [11, 29]. In addition, AMPK activity can inhibit fatty acid synthesis and cholesterol by lowering of FAS, SREBP-1c, and ACC as our data. These results demonstrate that DSR330 treatment negatively regulates the expression of lipogenesis-related proteins, including SREBP-1c and FAS, in the liver tissues of HFD-fed mice. SREBP-1c is an important transcription factor that regulates fatty acid, cholesterol, and TG synthesis, whereas FAS is involved in lipid accumulation [30]. Multi-strain probiotics, including *Bifidobacterium longum* LC67 and *L. plantarum* LC67, have been reported to alleviate liver steatosis in HFD-fed mice [31]. These strains regulate the activation of NF-κB and AMPK.

Some LABs have been reported to exert antiobesity effects via modulation of the metabolic pathways [32]. DSR330 mitigated changes in leptin, insulin, and adiponectin levels (Table 4). The NAFLD model showed increased leptin and insulin levels and decreased adiponectin levels. Leptin shows anti-steatosis effects in the early stages of NAFLD, which are mediated via fatty acid oxidation and a reduction in lipogenesis. Furthermore, it shows proinflammatory and pro-fibrotic effects at later disease stages by increasing hepatic reactive oxygen species generation and proinflammatory cytokine release and enhancing fibrinogenesis. Adiponectin regulates several metabolic functions, including glucose control and fatty acid oxidation. A reduction in adipocyte differentiation and an increase in energy expenditure associated with mitochondrial uncoupling is attributed to increased blood adiponectin concentrations [33, 34].

In summary, DSR330 showed hepatoprotective effects against NAFLD in HFD-fed mice. DSR330 significantly decreased body weight and liver weight and altered serum and hepatic lipid profiles, including a reduction in triglyceride, total cholesterol, and LDL cholesterol levels. DSR330 significantly reduced the levels of HFD-related hepatic injury markers, including AST, ALT, and ALP. Additionally, DSR330 downregulated the expression of SREBP-1c and FAS and upregulated the expression of ACC1. In addition, the levels of ACO, PPARα, and CPT-1 related to lipolysis and fatty oxidation were increased. Hormones related to energy metabolism were modulated by DSR330. These results demonstrate that *L. plantarum* DSR330 inhibited HFD-induced hepatic steatosis in mice with NAFLD by modulating signaling pathways and hormones, suggesting hepatoprotective effects of probiotics.

## Figures and Tables

**Table 1:** Effects of *Lactiplantibacillus plantarum* DSR330 on the body fat of HFD-fed mice.

Variable	ND	HFD	Silymarin	DSR-8	DSR-9
Relative adipose tissue weight (g/100 g BW)					
Brown fat	0.40 ± 0.05^b^	0.30 ± 0.03^ab^	0.27 ± 0.05^ab^	0.37 ± 0.04^ab^	0.28 ± 0.04^ab^
Subcutaneous fat	1.02 ± 0.16^a^	2.62 ± 0.49^c^	1.29 ± 0.28^ab^	2.42 ± 0.31^bc^	2.04 ± 0.27^ab^
Visceral fat	4.09 ± 0.31^a^	7.66 ± 0.73^b^	6.41 ± 1.03^b^	8.53 ± 0.81^b^	6.82 ± 0.76^b^
Epididymal fat	2.31 ± 0.17^a^	4.28 ± 0.37^b^	4.18 ± 0.58^b^	5.17 ± 0.46^b^	4.26 ± 0.45^b^
Retroperitioneal and perirenal	0.57 ± 0.11^a^	1.93 ± 0.29^ab^	1.14 ± 0.36^ab^	1.61 ± 0.29^ab^	1.37 ± 0.29^ab^
Mesenteric fat	1.22 ± 0.23^ns^	1.45 ± 0.35	1.10 ± 0.34	1.74 ± 0.56	1.19 ± 0.31

ND, normal diet (10% kcal fat); HFD, high-fat diet (60% kcal fat); Silymarin, HFD with silymarin; DSR-8, HFD with 10^8^ CFU/day of DSR330; DSR-9, HFD with 10^9^ CFU/day of DSR330.

Values represent mean ± standard error.

Values with different letters indicate significant differences calculated at *p* < 0.05 according to Duncan's multiple-range test.

^ns^not significant.

**Table 2:** Effect of *Lactiplantibacillus plantarum* DSR330 on relative gene expression associated with adipocyte differentiation and fat synthesis in HFD-fed mice.

Variable	ND	HFD	Silymarin	DSR-8	DSR-9
PPARγ	1.07 ± 0.06^bc^	1.55 ± 0.33^c^	0.31 ± 0.05^a^	1.42 ± 0.19^c^	0.80 ± 0.08^ab^
SREBP-1c	1.70 ± 0.14^a^	12.29 ± 0.4^8c^	4.49 ± 0.75^b^	12.35 ± 0.9^0c^	4.39 ± 0.49^b^
ACC1	1.15 ± 0.08^c^	0.18 ± 0.06^a^	0.59 ± 0.17^b^	0.93 ± 0.13^bc^	0.66 ± 0.09^b^
FAS	1.09 ± 0.01^ab^	4.86 ± 0.37^d^	0.94 ± 0.33^ab^	1.56 ± 0.20^c^	0.66 ± 0.01^a^

ND, normal diet (10% kcal fat); HFD, high-fat diet (60% kcal fat); Silymarin, HFD with silymarin; DSR-8, HFD with 10^8^ CFU/day of DSR330; DSR-9, HFD with 10^9^ CFU/day of DSR330.

Values represent the mean ± standard error.

Values with different letters indicate significant differences calculated at *p* < 0.05 according to Duncan's multiple-range test.

**Table 3:** Effect of *Lactiplantibacillus plantarum* DSR330 on relative gene expression associated with lipolysis and fatty oxidation in liver tissue.

Variable	ND	HFD	Silymarin	DSR-8	DSR-9
ACO	1.05 ± 0.03^b^	0.48 ± 0.10^a^	0.87 ± 0.12^b^	1.60 ± 0.13^c^	0.90 ± 0.13^b^
PPARα	1.18 ± 0.02^d^	0.52 ± 0.02^a^	0.70 ± 0.03^b^	0.96 ± 0.05^c^	0.69 ± 0.06^b^
CPT-1	1.09 ± 0.05^c^	0.25 ± 0.02^a^	0.50 ± 0.11^b^	0.91 ± 0.08^c^	0.68 ± 0.07^b^

ND, normal diet (10% kcal fat); HFD, high-fat diet (60% kcal fat); Silymarin, HFD with silymarin; DSR-8, HFD with 10^8^ CFU/day of DSR330; DSR-9, HFD with 10^9^ CFU/day of DSR330.

Values represent the mean ± standard error.

Values with different letters indicate significant differences calculated at *p* < 0.05 according to Duncan's multiple-range test.

**Table 4:** Effect of *Lactiplantibacillus plantarum* DSR330 on hormone related to serum energy metabolism.

Variable	ND	HFD	Silymarin	DSR-8	DSR-9
Insulin (ng/ml)	1.65 ± 0.35^a^	11.61 ± 0.19^c^	2.45 ± 0.25^b^	2.73 ± 0.13^b^	2.51 ± 0.34^b^
Adiponectin (μg/ml)	28.72 ± 1.87^b^	13.62 ± 1.85^a^	25.23 ± 2.74^b^	29.19 ± 0.79^b^	29.95 ± 0.79^b^
Leptin (ng/ml)	13.98 ± 0.54^a^	75.61 ± 0.94^e^	24.66 ± 1.42^b^	43.29 ± 1.21^c^	46.83 ± 1.46^d^

ND, normal diet (10% kcal fat); HFD, high-fat diet (60% kcal fat); Silymarin, HFD with silymarin; DSR-8, HFD with 10^8^ CFU/day of DSR330; DSR-9, HFD with 10^9^ CFU/day of DSR330.

Values are the mean ± standard error.

Values with different letters are significantly different at *p* < 0.05, according to Duncan's multiple-range test.
